# Factor Structure of the Cannabis Use Disorders Identification Test Revised (CUDIT-R) for Men and Women

**DOI:** 10.26828/cannabis.2020.02.002

**Published:** 2020-07-03

**Authors:** Megan M. Risi, Alexander W. Sokolovsky, Helene R. White, Kristina M. Jackson

**Affiliations:** 1 Department of Psychology, University of Rhode Island, South Kingston, RI, USA; 2 Center for Alcohol and Addiction Studies, School of Public Health, Brown University, Providence, RI, USA; 3 Department of Sociology and Center of Alcohol Studies, Rutgers, the State University of New Jersey, Piscataway, NJ, USA

**Keywords:** cannabis, CUDIT-R, college students, assessment

## Abstract

The Cannabis Use Disorders Identification Test Revised (CUDIT-R) is an 8-item screening instrument designed to identify recent problematic cannabis use over the past 6 months. The purpose of the present study was to investigate the factor structure of the CUDIT-R separately for male and female college students. Participants included 1,390 male and female college students recruited from three state universities (61% female; Age: *M*= 19.8, *SD*= 1.3). We conducted exploratory and confirmatory factor analyses followed by tests of measurement invariance including configural invariance, metric invariance and scalar invariance across men and women. Results confirmed a one-factor structure for the CUDIT-R. The number of factors and item loadings were invariant between men and women. However, intercepts were non-invariant for an item asking about consumption of cannabis use indicating that the endorsement of this item varied between men and women. Follow-up validation tests indicated that using a sum score for analyses is appropriate despite non-invariance. However, more research is needed to determine if the cut-off scores of the CUDIT-R should be reevaluated by gender.

The 20-item Cannabis Use Disorders Identification Test (CUDIT; Adamson & Sellman, 2003) was developed to screen for cannabis abuse or dependence by modifying the Alcohol Use Disorders Test (AUDIT; Saunders, Aasland, Babor, De la Fuente, & Grant, 1993). The initial CUDIT was revised (CUDIT-R; Adamson et al, 2010) and contains eight items consisting of four items from the original CUDIT and four new items that assess consumption, problems, dependence, and psychological features. Although the CUDIT-R has been validated in clinical (Adamson et al., 2010) and non-clinical (Loflin, Babson, Browne, & Bonn-Miller, 2018; Schultz, Bassett, Messina, & Correia, 2019) samples, it is not known whether the CUDIT-R operates differently across salient demographic groups, such as men and women.

Men and women differ in rates of cannabis use and progression to cannabis use disorder (CUD). Reporting of lifetime cannabis use is about 53.1% in men and 43.7% in women (Substance Abuse and Mental Health Services Administration, 2018). Recent research indicates that men also have higher rates of CUD than women (3.5% versus 1.7%, respectively; Hasin et al, 2016). Nonetheless, women appear to have a faster trajectory from first cannabis use to CUD relative to men (Hernandez-Avila, Rounsaville, & Kranzler, 2004; Khan, 2013). The reason for this telescoping effect is not well known; however, women demonstrate greater subjective intoxication to cannabis than men, which may contribute to maintained use (Cooper & Haney, 2014).

The validity of the CUDIT-R across gender has not, to our knowledge, been investigated. Thus, current research utilizing the CUDIT-R relies on the assumption that this measure assesses the same construct in men and women across a common metric. Without examining measurement invariance, we do not have evidence that differences among men and women in the CUDIT-R scores represent true differences in problematic use or are merely artifacts of other processes, such as the interpretation of questions. The purpose of the present study was to explore the psychometric qualities of the CUDIT-R separately for young adult men and women.

## METHOD

### Participants and Procedures

As part of a larger study, students ages 18-24 from three state universities were randomly chosen by each school’s registrar and sent email invitations to participate in an online screening survey. Eligible participants were then invited to participate in a 30- to 40-minute online baseline survey asking questions related to alcohol and cannabis use. Eligibility criteria included being a past-year alcohol and cannabis user between ages 18 and 24 and a full-time student invited to participate at one of the three universities. Those who responded to the screening surveys were fairly representative of those invited based on demographic information provided by the registrars. (For greater detail on recruitment and participation, see White et al., 2019.)

Participants were 1,390 eligible students from the three universities. Gender identity of the sample was 61% women, 38.1% men, and 0.9% transgender, genderqueer, or gender non-binary, with a mean age of 19.8 (*SD* = 1.3). The sample was 63.8% non-Hispanic White, 2.7% non-Hispanic Black, 12.5% Asian, 12.2% Hispanic/Latinx, 0.2% Native Hawaiian or other Pacific Islander, 0.1% American Indian or Alaskan Native, 0.8% other not listed, and 7.7% more than one race/ethnicity.

### Measures

The CUDIT-R (Adamson et al, 2010) contains items designed to assess criteria related to cannabis abuse and dependence during the past 6 months and has been validated to screen for DSM-5 criteria for CUD (Schultz et al., 2019). See Table 1 for questions and item-level means. Item one had response options of 0 = “Never,” 1 = “Monthly or less,” 2 = “2-4 times a month,” 3 = “2-3 times a week,” and 4 = “4 or more times a week.” Item eight used response options 0 = “Never,” 2 = “Yes, but not in the past 6 months,” and 4 = “Yes, during the past 6 months.” The remaining six items used a five-point Likert-type scale ranging from 0 = “Never” to 4 = “Daily or almost daily.” Total scores range from 0 to 32 with scores of 8 or more indicating hazardous cannabis use and scores of 12 or more indicating possible CUD (Adamson et al., 2010). In addition, demographic information including gender identity was collected. Response options included Male, Female, Tans male/Trans man, Trans female/Trans woman, Genderqueer/Gender non-conforming, different identity (check all that apply). For the analyses, we included persons who identify as either men or women. Thus, those who identified as transgender men were combined with those who identified as men (total *n* = 547) and those who identified as transgender women were combined with women (total *n* = 831). We excluded participants who identified as genderqueer and gender nonbinary if they did not also identify as a man or woman as the sample size was too small (*n* = 9). Further, we excluded participants who identified as both a man and woman (*n* = 3). The final sample size for the EFA was 488 (men *n* = 199, women *n* = 289) and the final sample for the CFA was 890 (men *n* = 348, women *n* = 542).

Students were asked whether or not they experienced 28 different negative consequences in the past 3 months “due to their marijuana use” (see Table 2). We summed these dichotomous items (yes/no) to create a score of total number of consequences experienced. The consequence items were from the 21-item Brief Marijuana Consequences Questionnaire (MACQ; Simons, Dvorak, Merrill, & Read, 2012) and the 24-item Brief Young Adult Alcohol Consequence Questionnaire (BYAACQ; Kahler, Strong, & Read, 2005); collapsing the two scales yielded 28 unique items. Both scales have been used reliably with college students (Kahler et al., 2005; Simons et al., 2012).

### Data Analysis

A random subset of 488 participants was first used for exploratory factor analysis (EFA), which was further split by gender. We conducted the EFA using R version 3.1.4 (R Core Team, 2017) on the CUDIT-R and factor extraction was based on parallel analysis (Hayton, Allen, & Scarpello, 2004). Factor analysis was justified using Bartlett’s test of sphericity and the Kaiser Meyer-Oklin (KMO) measure of sampling adequacy (Bartlett, 1950; Kaiser, 1970). A significant Bartlett’s test (p<.05) and a KMO index of at least 0.50 indicated the data were suitable for factor analysis (Williams, Onsman, & Brown, 2010). The remaining 890 participants were used to conduct the CFA and measurement invariance.

Table 1 shows descriptive statistics for individual CUDIT-R items. The CFA was completed using lavaan (Rosseel, 2012) for R version 3.1.4 (R Core Team, 2017). Missing data were accounted for using Diagonally Weighted Least Squares (DWLS), which results in less biased factor loadings for ordinal data (Li, 2016). The Comparative Fit Index (CFI) and Tucker Lewis Index (TLI) ≥.95, the Root Mean Square Error of Approximation (RMSEA) ≤ .06, and the standardized root mean squared residual (SRMR) ≤ .08 were used as indicators for good model fit (Hu & Bentler, 1999; Yu, 2002). Modification indices were evaluated to determine whether residuals of items should be correlated based on overlapping constructs (Sörbom, 1989).

Next, measurement invariance was tested by sequentially constraining parameters across genders. Configural invariance of the CUDIT-R was evaluated by first fitting separate confirmatory models in men and women. A test of configural invariance examines whether the basic organization of the constructs (i.e., latent factors) is supported across genders. Once configural invariance is established, the next step is metric invariance, or invariance of the item loadings. When factor loadings are invariant across groups, this indicates that each item contributes to the latent construct to a similar degree. If metric invariance holds across groups, scalar invariance is tested. Scalar invariance is the equivalence of item intercepts. If all previous invariances are supported, strict invariance is tested. Strict, or residual, invariance tests whether the sum of specific variance and error variance is similar across groups (Byrne, 2010; Kline, 2011 Putnick & Bornstein, 2016). The marijuana consequences score was used to validate the CUDIT-R.

## RESULTS

The criteria of sphericity and normality were met as checked by a significant Bartlett’s sphericity test (p<.001) and KMO value of 0.85. Parallel analysis and EFA suggested a one-factor solution for the CUDIT-R for both men and women with 83% of variance explained for both samples (see Table 3).

Next, we tested goodness of fit of the one-factor structure using CFA. The final sample for the CFA (*N* = 890) consisted of 348 men and 542 women. There was no significant difference in gender by site, χ^2^(2) = 1.36, *p* = .506. A one-factor model with no correlated residuals showed poor to adequate fit (i.e., χ^2^(20) = 92.431, *p* < .001, CFI = .964, TLI = .950, RMSEA[90%CI] = .064[.051, .077], SRMR = .034). Evaluation of the modification indices showed strong evidence of a correlated residual between item one (“How often do you use cannabis?”) and item seven (“How often do you use cannabis in situations that could be physically hazardous, such as driving, operating machinery, or caring for children?”). Given that these two items tapped similar content (frequency of use), we made the decision to correlate the residuals. The model with these correlated residuals resulted in significant improvement in model fit, Δχ^2^ = 21.597, Δdf = 1, *p* < .001. Modification indices were reevaluated and suggested that the covariance of item one and item two (“How many hours were you ‘stoned’ on a typical day when you had been using cannabis?”) also overlapped, likely because both items are indicators of consumption (as opposed to problems). Adding these correlated residuals resulted in significant improvement in model fit, Δχ^2^= 21.229, Δdf = 1, *p* < .001.

Modification indices were reevaluated once again but suggested no correlations with overlapping constructs. The final model showed good to excellent fit, χ^2^(17) = 49.605, *p* < .001, CFI = .984, TLI = 0.976, RMSEA[90%CI] = .044[.030, .059], SRMR = 0.025.

**Figure 1.**
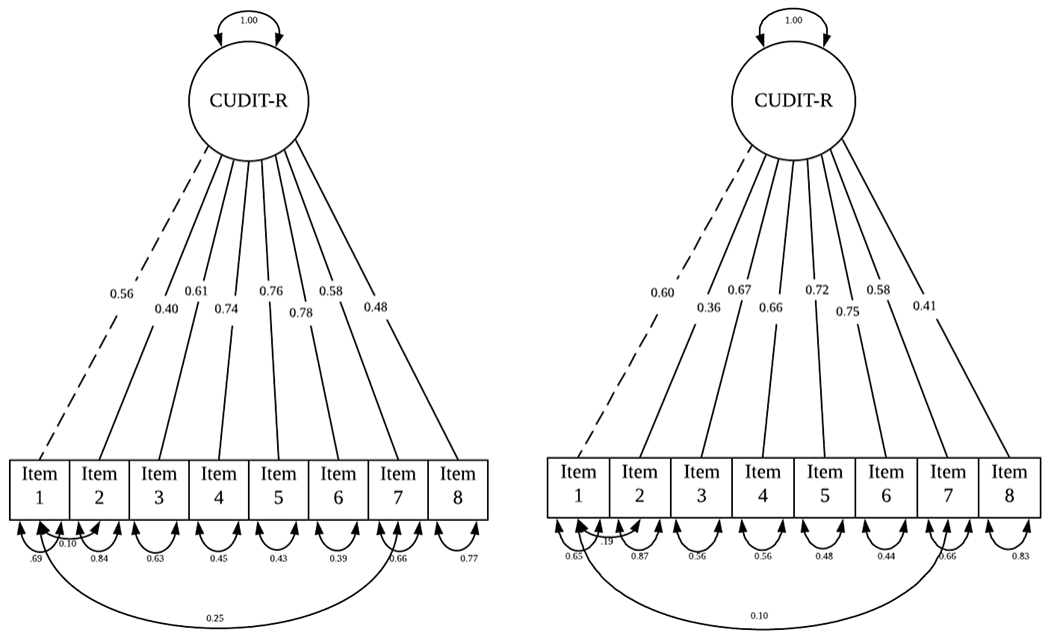
Model with standardized estimates for men (left) and women (right) separately.

The final one-factor CFA model was run for men and women separately. The one-factor CFA showed good fit for men, χ^2^(18) = 44.403, *p* = .001, CFI = .969, TLI = 0.951, RMSEA[90%CI] = .066[.041, .090], SRMR = 0.039; and women, χ^2^(18) = 36.489, *p* = .006, CFI = .984, TLI = 0.975, RMSEA[90%CI] = .026[.022, .063], SRMR = 0.026 (see Figure 1 final model by gender).

Table 4 shows the results from invariance testing. After establishing configural invariance, we tested group invariance by entering the configural model as the baseline step (Step 1), and constraining factor loadings to be equal across groups (metric invariance; Step 2). We found that the strengths of the factor loadings were invariant across men and women. We then evaluated scalar invariance by further constraining item intercepts to be equal across groups (Step 3). We found non-invariant intercepts for item one suggesting that scalar invariance did not hold between men and women. Specifically, men had a higher unstandardized item intercept than women (intercept = 2.91, *SE*= 0.083 and intercept = 2.41, *SE* = 0.062, respectively).

We further evaluated whether a sum score for the CUDIT-R reliably indexed the measure for both men and women, by computing factor scores for each individual and correlating this score with the CUDIT-R sum score. Pearson correlation revealed that the CUDIT-R factor score was strongly correlated with the sum score (*r* = .991, *p* < .001), suggesting that the variance in these indices was largely overlapping. Finally, to determine whether differences observed in our test of scalar invariance would have practical implications for the CUDIT-R at a substantive level, we investigated the concurrent validity of both index measures (sum scores and factor scores) and found that they were both significantly correlated with cannabis use consequences with relatively equivalent magnitude (*r*= .711 , *p* < .001 and *r* = .711, *p* < .001, respectively). When split by sex, both index measures correlated significantly with men (*r* = .684, *p* < .001 and *r* = .689, *p* < .001, respectively) and women (*r* = .724, *p* < .001 and *r* = .720, *p* < .001, respectively).

## DISCUSSION

The present study sought to replicate the factor structure of the CUDIT-R items proposed by Adamson et al. (2010) in a nonclinical young adult sample of cannabis users and to extend previous studies by examining gender invariance in the CUDIT-R. In line with the conceptualization of the CUDIT-R, our model confirmed a one-factor structure. Our test of whether the CUDIT-R factor structure was the same across men and women indicated that the number of factors and item loadings were invariant between men and women.

Although our model replicated the factor structure of the CUDIT-R, we used modification indices to identify items with shared variance. Specifically, item one (“How often do you use cannabis”) was correlated with items two (“How many hours were you ‘stoned’ on a typical day when you had been using cannabis”) and seven (“How often do you use cannabis in situations that could be physically hazardous, such as driving, operating machinery, or caring for children”). These items strongly overlapped in asking about consumption patterns. Invariance testing of the CUDIT-R held across factor loadings (i.e., metric invariance). This finding indicates that the relationship between CUDIT-R items and the underlying latent construct is the same for men and women and suggests that these items are interpreted consistently by both genders.

In this sample, item one intercept (“How often do you use cannabis?”) was non-invariant across groups, indicating that the endorsement of the items varied between men and women. Knowing that there are gender differences in cannabis use including prevalence (Cuttler, Mischley, & Sexton, 2016) and rates of and progression to CUD (Hasin et al., 2016; Hernandez-Avila et al., 2004; Khan, 2013), differences in endorsement of CUDIT-R items was expected. Specifically, we found that men had higher endorsement of item one (“How often do you use cannabis?”). This difference is in line with previous research indicating that men use cannabis more frequently than women (Cuttler et al., 2016).

Due to the gender differences in the CUDIT-R above, we compared the traditionally derived CUDIT-R sum score to a CUDIT-R factor score based on our psychometric models. Factor scores are composite scores which identify an individual’s placement on a latent factor. When we compared the factor score with the sum score, results indicated both scores measured virtually the same thing (i.e., they were correlated at .99). This finding suggests that despite non-invariance at item one intercepts, the sum score is still appropriate to use for both young adult men and women. However, more research is needed to determine if clinical implications of the CUDIT-R, such as cut-off scores, should be reevaluated by gender.

The results of the study need to be considered within the context of some limitations. The CUDIT-R is a self-report measure; thus, responses may be over- or under-reported. The present study results were based on a sample of university students who reported using both alcohol and cannabis in the past year and may not generalize to other college students or to non-student samples. The CUDIT-R may perform differently in other samples such as older adults or those with less regular cannabis use who may endorse items related to frequency at lower levels. Nonetheless, our sample represents an important age group given that the annual prevalence of cannabis is highest among 19- to 30-year-olds (38%) with highest use at ages 21-22 (44%; Schulenberg et al, 2019) and odds of CUD diagnosis are highest in young adults aged 18-24 (Hasin et al., 2016). Our sample had a small number of non-white students and analyses were limited to those who identified either as men or women; replication in more diverse samples and across non-binary gender groups is an area of future research. Due to the self-report nature of the assessment and the lack of a diagnostic measure of CUD in the data set, we were unable to determine potential cutoff scores for hazardous use and probable CUD. Future research should work to determine appropriate cutoff scores for men and women.

Despite these limitations, this is the first study that has evaluated the factor structure of the CUDIT-R separately for men and women. This study makes a significant contribution through the evaluation of this screening tool across genders, which could have clinical implications for the identification of problematic cannabis use and CUD. With recent legislative changes in cannabis legalization as well as increased prevalence of cannabis use, identifying problematic use will be imperative.

## Figures and Tables

**Table 1.** Descriptive information for CUDIT-R (N = 1390)

Item	Mean (*SD*)	Range
Frequency of cannabis use	1.32 (1.54)	1-5
Hours stoned on a typical day	1.32 (0.88)	1-5
Past 6 months unable to stop using cannabis once started	0.23 (0.73)	1-5
Past 6 months failed to do what’s expected because of cannabis use	0.35 (0.69)	1-5
Past 6 months devoted time spent recovering from cannabis use	0.34 (0.82)	1-5
Past 6 months problem with memory or concentrating because of cannabis use	0.59 (0.94)	1-5
Cannabis use in risky or hazardous situation	0.43 (0.89)	1-5
Thought about cutting down or stopping cannabis use	1.82 (1.89)	1-3

*Note*. The CUDIT-R asks “How often in the past 6 months have you…”; Item one had response options of 0=“Never,” 1=“Monthly or less,” 2=“2-4 times a month,” 3=“2-3 times a week,” and 4=“4 or more times a week.” Item eight used response options 0=“Never,” 2=“Yes, but not in the past 6 months,” and 4=“Yes, during the past 6 months.” The remaining six items had responses of 0=“Never,” 1=“Less than monthly, 2=“Monthly,” 3=“Weekly,” and 4=“Daily or almost daily.” *SD* = Standard Deviation

**Table 2.** Negative Consequences for Cannabis Use Experienced Over the Past 3 Months

Item
1. Had a hangover or felt in a fog the morning after I had been using
2. My school work has suffered because of my use
3. I had less energy or felt tired because of my use
4. Have often ended up using on nights when I had planned not to use
5. While using, I have said or done embarrassing things
6. Have missed classes because of use, hangover, or illness caused by my use
7. When using, I have done impulsive things I regretted later
8. My use has created problems between myself and my romantic partner or parents
9. Have felt like I needed to use after I’d gotten up (i.e., before breakfast)
10. Have neglected my obligations to family, work, or school because of my use
11. Have often found it difficult to limit how much I use
12. Have become very rude, obnoxious, or insulting after use
13. Have felt very sick to my stomach or thrown up after using
14. Have taken foolish risks when I have been using
15. Have passed out from using
16. Could no longer get high on the amount that used to get me high
17. My use has gotten me into sexual situations that I later regretted
18. Have woken up in an unexpected place after using heavily
19. Have driven a car while under the influence
20. Have gotten into physical fights because of my use
21. Have been less physically active because of my use
22. Have had trouble sleeping after stopping or cutting down on use
23. Have awakened the day after using and found I could not remember a part of the evening before
24. Haven’t been as sharp mentally because of my use
25. Have received a lower grade on an exam or paper than I normally would have because of my use
26. Have tried to quit using because I thought I was using too much
27. Have felt anxious, irritable, lost my appetite or had stomach pains after stopping or cutting down use
28. Have lost motivation to do things because of my use

Note. All questions were asked as yes or no

**Table 3.** Factor Loadings of the Individual CUDIT-R Items on the Factor for Men and Women Separately from Exploratory Factor Analysis.

Item	Men (*N* = 199)	Woman (*N* = 289)
Frequency of cannabis use	0.59	0.66
Hours stoned on a typical day	0.31	0.39
Past 6 months unable to stop using cannabis once started	0.62	0.58
Past 6 months failed to do what’s expected because of cannabis use	0.56	0.52
Past 6 months devoted time spent recovering from cannabis use	0.65	0.62
Past 6 months problem with memory or concentrating because of cannabis use	0.74	0.81
Cannabis use in risky or hazardous situation	0.63	0.48
Thought about cutting down or stopping cannabis use	0.53	0.57

*Note.* The CUDIT-R asks “How often in the past 6 months have you…”; Item one had response options of 0=“Never,” 1=“Monthly or less,” 2=“2-4 times a month,” 3=“2-3 times a week,” and 4=“4 or more times a week.” Item eight used response options 0=“Never,” 2=“Yes, but not in the past 6 months,” and 4=“Yes, during the past 6 months.” The remaining six items had responses of 0=“Never,” 1=“Less than monthly, 2=“Monthly,” 3=“Weekly,” and 4=“Daily or almost daily.”

**Table 4.** Invariance Testing Across Gender for the One-Factor Model of the CUDIT-R (N = 890)

Model	χ^2^(df)	CFI	RMSEA	Δχ^2^(Δdf)	ΔCFI	*p*
Step 1: Configural model	80.270 (36)	.978	.053	-	-	-
Step 2: Metric Invariance (all factor loadings held invariant across groups)	85.998 (43)	.979	.047	5.728 (7)	0.001	0.572
Step 3: Scalar Invariance (all factor loadings and item intercepts held invariant across groups)	134.182 (50)	.958	.062	48.184 (7)	0.020	< .001

## References

[R1] Adamson SJ & Sellman JD (2003). A prototype screening instrument for cannabis use disorder: The Cannabis Use Disorders Identification Test (CUDIT) in an alcohol dependent clinical sample. Drug and Alcohol Review, 22(3), 309–315. DOI: 10.1080/0959523031000154454 15385225

[R2] Adamson SJ, Kay-Lambkin FJ, Baker AL, Lewin TJ, Thornton L, Kelly BJ, & Sellman JD (2010). An improved brief emasyre of cannabis misuse: The Cannabis Use Disorders Identification Test-Revised (CUDIT-R). Drug and Alcohol Dependence, 110(1-2), 137–143. DOI: 10.1016/j.drugalcdep.2010.02.017 20347232

[R3] Bartlett MS (1950). Tests of significance in factor analysis. British Journal of Statistical Psychology, 3(2), 77–85. DOI: 10.1111/j.2044-8317.1950.tb00285.x

[R4] Byrne BM (2012). A primer of LISREL: Basic applications and programming for confirmatory factor analytic models. Springer Science & Business Media.

[R5] Caulkins JP, Kilmer B, Kleiman MAR, MacCoun RJ, Midgette G, Oglesby P,…, Reuter PH (2015). The Marijuana Legalization Debate, RAND Corporation: SantaMonica, CA.

[R6] Cole DA, Ciesla JA, & Steiger JH (2007). The insideous effects of failing to include design-driven correlated residuals in latent-variable covarate structure analysis. Psychological Methods, 12(4), 381–298. DOI: 10.1037/1082-989X.12.4.381 18179350

[R7] Cooper ZD & Haney M (2014). Investigation of sex-dependent effects of cannabis in daily cannabis smokers. Drug and Alchol Dependence, 136, 85–91. DOI: 10.1016/j.drugalcdep.2013.12.013 PMC4518446 24440051

[R8] Cuttler C, Mischley LK, & Sexton M (2016). Sex differences in cannabis use and effects: A cross-sectional survey of cannabis users. Cannabis and Cannabinoid Research, 1(1), 166–175. DOI: 10.1089/can.2016.0010 28861492 PMC5576608

[R9] Hasin DS, Kerridge BT, Saha TD, Huang B, Pickering R, Smith SM … Grant BF (2016) Prevalence and correlates of DSM-5 cannabis use disorder, 2012-2013: findings from the national epidemiologic survey on alcohol and related conditions-III The American Journal of Psychiatry, 173(6), 588–599. DOI: 10.1176/appi.ajp.2015.15070907 26940807 PMC5026387

[R10] Hayton JC, Allen DG, & Scarpello V (2004). Factor retention decisions in exploratory factor analysis: A tutorial on parallel analysis. Organizational Research Methods, 7(2), 191–205. DOI: 01177/1094428104263675

[R11] Hernandez-Avila CA, Rounsaville BJ, & Kranzler HR (2004). Opioid-, cannabis- and alcohol-dependent women show more rapid progression to substance abuse treatment. Drug and Alcohol Dependence, 74, 265–272. DOI: 10.1016/j.drugalcdep.2004.02.001 15194204

[R12] Kahler CW, Strong DR, & Read JP (2005) Toward efficient and comprehensive measurement of the alcohol problems continuum in college students: The Brief Young Adult Alcohol Consequences Questionnaire. Alcoholism: Clinical and Experimental Research, 29, 1180–1189. DOI: 10.1097/01.ALC.000017940.95813.A5 16046873

[R13] Kaiser HF (1970). A second-generation little jiffy. Psykometrika, 35(4), 401–415. DOI: 10.1007/BF02291817

[R14] Khan SS, Secades-Villa R, Okuda M, Wang S, Pérez-Fuentes G, Kerridge BT, & Blanco C (2013). Gender differencces in cannabis use disorders: Results from the National Epidemiologic Survey of Alcohol and Related Conditions. Drug and Alcohol Dependence, 130, 101–108. DOI: 10.1016/drugalcdep.2012.10.015 23182839 PMC3586748

[R15] Kline RB (2011). Methodology in the Social Sciences. Principles and practices of structural equation modeling (3rd ed.). New York, NY, US: Guilford Press.

[R16] Li CH (2016). The performance of ML, DWLS, and ULS estimation with robust corrections in structural equation models with ordinal variables. Psychological Methods, 21(3), 369–387. DOI: 10.1037.met0000093 27571021 10.1037/met0000093

[R17] Loflin M, Babson K, Browne K, & Bonn-Miller M (2018). Assessment of the validity of the CUDIT-R in a subpopulation of cannabis users. The American Journal of Alcohol Abuse, 44(1), 19–23. DOI: 10.1080/00952990.2017.136677 29058471

[R18] Putnick DL & Bornstein MH (2016).Measurement invariance conventions and reporting: The state of the art and future directions for psychological research. Developmental Review, 41, 71–90. DOI: 10.1016/j.dr.2016.06.004 27942093 PMC5145197

[R19] R Core Team (2017). R: A language and environment for statistical computing. R Foundation for Statistical Computing, Vienna, Austria.

[R20] Rosseel Y (2012). lavaan: An R package for structural equation modeling and more. Journal of Statistical Software, 48(2), 1–36. DOI: 10.18637/jss.v048.i02

[R21] Saunders JB, Aasland OG, Babor TF, De la Fuente JR, & Grant M (1993). Development of the alcohol use disorders identification test (AUDIT): WHO collaviraruce orihecr ib early detection of persons with harmful alcohol consumption-II. Addiction, 88(6), 791–804. DOI: 10.1111/j.1360-0443.1993.tb02093.x 8329970

[R22] Schulenberg JE, Johnston LD, O’Malley PM, Bachman JG, Miech RA, & Patrick ME (2019). Monitoring the Future national survey results on drug use, 1975-2018: Volume II, College students and adults ages 19-60. Ann Arbor: Institute for social research, The University of Michigan. Available at http://monitoringthefuture.org/pubs.html#monographs

[R23] Schultz NR, Bassett DT, Messina BG, & Correia CJ (2019). Evaluation of the psychometric properties of the Cannabis Use Disorders Identification Test-Revised among college students. Addictive Behaviors, 95, 11–15. DOI: 10.1016/j.addbeh.2019.02.016 30798191

[R24] Simons JS, Dvorak RD, Merrill JE, & Read JP (2012). Dimensions and severity of marijuana consequences: Development and validation of the Marijuana Consequences Questionnaire (MACQ). Addictive Behaviors, 37(5), 613–621. DOI: 10.1016/j.addbeh.2012.01.008 22305645 PMC3307958

[R25] Sörbom D (1989). Model modification. Psychometrika, 54(3), 371–384.

[R26] Substance Abuse and Mental Health Services Administration (2018). 2017 National Survey on Drug Use and Health: Detailed Tables. Center for Behavioral Health Statistics and Quality, Rockville, MD.

[R27] White HR, Kilmer JR, Fossos-Wong N, Hayes K, Sokolovsky AW, & Jackson KM (2019). Simultaneous alcohol and marijuana use among college students: Patterns, correlates, norms, and consequences. Alcoholism: Clinical and Experimental Research, 43(7), 1545–1555. DOI: 10.1111/acer.14072 31135972 PMC6640138

[R28] Williams B, Onsman A, & Brown T (2010). Exploratory factor analysis: A five-step guide for novices. Journal of Emergency Primary Health Care, 8(3), 633–643. DOI: 10.33151/ajp.8.3.93

[R29] Yu CY (2002). Evaluating cutoff criteria of model fit indices for latent variable models with binary and continuous outcomes (Vol. 30). Los Angeles: University of California, Los Angeles. DOI: 10.1.1.310.3956

